# Analysis of Bi Distribution in Epitaxial GaAsBi by Aberration-Corrected HAADF-STEM

**DOI:** 10.1186/s11671-018-2530-5

**Published:** 2018-04-25

**Authors:** N. Baladés, D. L. Sales, M. Herrera, C. H. Tan, Y. Liu, R. D. Richards, S. I. Molina

**Affiliations:** 1 Department of Material Science, Metallurgical Engineering and Inorganic Chemistry, University of Cádiz, Campus Río San Pedro, 11510, Puerto Real, Cádiz, Spain; 2 Instituto Universitario de Investigación en Microscopía Electrónica y Materiales (IMEYMAT). Facultad de Ciencias. Universidad de Cádiz, Campus Río San Pedro s/n. 11510 Puerto Real, Cádiz, Spain; 3 Department of Electronic and Electrical Engineering, University of Sheffield, S3 7HQ, Sheffield, UK

**Keywords:** GaAsBi, Ac-HAADF-STEM, Bi-clusters

## Abstract

The Bi content in GaAs/GaAs_1 − *x*_Bi_*x*_/GaAs heterostructures grown by molecular beam epitaxy at a substrate temperature close to 340 °C is investigated by aberration-corrected high-angle annular dark-field techniques. The analysis at low magnification of high-angle annular dark-field scanning transmission electron microscopy images, corroborated by EDX analysis, revealed planar defect-free layers and a non-homogeneous Bi distribution at the interfaces and within the GaAsBi layer. At high magnification, the qHAADF analysis confirmed the inhomogeneous distribution and Bi segregation at the GaAsBi/GaAs interface at low Bi flux and distorted dumbbell shape in areas with higher Bi content. At higher Bi flux, the size of the Bi gathering increases leading to roughly equiaxial Bi-rich particles faceted along zinc blende {111} and uniformly dispersed around the matrix and interfaces. FFT analysis checks the coexistence of two phases in some clusters: a rhombohedral pure Bi (rh-Bi) one surrounded by a zinc blende GaAs_1 − *x*_Bi_*x*_ matrix. Clusters may be affecting to the local lattice relaxation and leading to a partially relaxed GaAsBi/GaAs system, in good agreement with XRD analysis.

## Background

Nowadays, GaAsBi-based semiconductors are attracting interest as temperature-stable and mid-infrared devices [1]. Adding a small amount of Bi into the GaAs lattice leads to a large bandgap reduction, being one of the most interesting optoelectronic effects [2, 3]. However, incorporating even a small amount of Bi into GaAs is challenging due to the weak Ga–Bi bonding energy, the large miscibility gap and the large difference in the lattice constant between GaBi and GaAs. Consequently, GaAsBi has to be grown under a non-equilibrium dynamic process for an efficient Bi incorporation. Although even if it is synthesised successfully, the distribution of Bi is sometimes non-uniform, increasing the density of non-radiative recombination centres and therefore affecting their efficiency in lasing operation. The large size and low electronegativity of Bi tend to produce phase separation [4], surface droplets [5, 6], atomic ordering [7–9], nano-scale liquid droplets [10] or Bi clusters during epitaxial growth. The presence of Bi clusters was previously detected by Ciatto et al. through a combination of X-ray absorption spectroscopy (XAS), atomic force microscopy and X-ray diffraction (XRD) techniques [11]. Then, several authors reported the presence of Bi clusters in annealed GaAsBi samples by using different transmission electron microscopy techniques [4, 12, 13]. Furthermore, Kunzer et al. [14] confirmed by conventional electron spin resonance (ESR) that about 10% of the incorporated Bi had occupied the Ga sites in GaAsBi layers. Therefore, understanding and controlling the Bi incorporation and defect formation is critical for the successful application of GaAsBi to devices. It is worth mentioning that the development of new Bi-based materials is linked to the advancement of characterization tools. In this sense, high-angle annular dark-field scanning transmission electron microscopy (HAADF-STEM) techniques in aberration-corrected microscopes plays an important role in obtaining information at sub-angstrom level [15, 16]. In this technique, the intensity in the images is roughly proportional to the average atomic number (*Z*) in the projected atomic column, so it can be successfully applied in dilute Ga(AsBi) heterostructures due to the large difference in the atomic number of Bi with respect to As and Ga. In addition, bright features in HAADF images, contrary to the high-resolution transmission electron microscopy (HRTEM), can be associated to atomic columns in an aligned crystal due to the lack of contrast reversals and delocalization. Also, aberration-corrected HAADF images of GaAsBi samples show a low dependence on the specimen thickness and an almost linear dependence on the As/Bi composition [12]. Moreover, by applying the quantitative HAADF (qHAADF) image analysis algorithm, developed by Molina et al. [17], it is possible to correlate effectively HAADF intensity and atomic column composition in III–V ternary semiconductor materials [12, 16, 18] and consequently in GaAsBi compounds. Additionally, high-resolution HAADF-STEM images offer information about crystalline quality [19–22].

In this work, we investigate through aberration-corrected HAADF-STEM imaging and complementary energy-dispersive X-ray (EDX) the Bi distribution in GaAs/GaAs_1 − *x*_Bi_*x*_/GaAs heterostructures grown by molecular beam epitaxy (MBE) at a substrate temperature close to 340 °C. We also studied the effect of the Bi gathering at nano- and micro-scales. For this aim, we have used the qHAADF approach, the Fourier fast transform (FFT) analysis of high-resolution images and the XRD techniques.

## Methods

We study a series of two samples consisting of GaAs/GaAs_1 − *x*_Bi_*x*_/GaAs heterostructures grown by solid source MBE on 2″ n^+^ GaAs: Si (001) wafers with different Bi flux given by the Bi cell temperatures S1 (460 °C) and S2 (505 °C). The system used was a VG V80 MBE machine with an average resting background pressure of ~ 5 × 10^− 10^ mbar. The structures nominally consist of a 130 nm GaAs buffer, 130 nm GaAsBi layer, 5 nm GaAs spacer layer and then a 130 nm GaAs cap. Prior to growth, the substrate was outgassed at 400 °C for 20 min and then the surface oxide was removed at 600 °C. The GaAs buffer and cap were grown at ~ 580 °C under an As_2_ flux, while the GaAsBi layer and GaAs spacer layer were grown at ~ 340 °C under a near-stoichiometric As_4_ flux. The GaAsBi layer was therefore annealed in situ at ~ 580 °C for ~ 20 min during the growth of the GaAs cap. Under these growth conditions, no metallic droplets at the surface were observed. The substrate temperatures were estimated using optical thermometry, with the results being calibrated against surface reconstruction transitions at known temperatures. Prior to GaAsBi growth, the sample surface was exposed to a Bi flux for 20 s; the purpose of this step was to establish a Bi surface layer and enhance Bi incorporation at the commencement of GaAsBi growth.

The specimens for HAADF-STEM were prepared in cross-section by mechanical grinding and Ar^+^ ion milling using a precision ion polishing system (PIPS), with beam tilts of − 3° and + 4° and beam energy between 2.8 and 3.0 kV. At the final milling step, the ion energy was decreased to 1.5 kV to enhance surface quality. Samples before being studied were plasma-cleaned to reduce the effect of the electron beam deposition on the specimen surface [23]. HAADF-STEM images, zero-loss electron energy loss spectrum (EELS) and energy-dispersive X-ray spectra line scans were conducted at 200 kV, using a Titan^3^ Themis at 60–300 kV. The Titan^3^ Themis is equipped with a cold field emission gun (FEG), Cs probe corrector and electron monochromator, allowing atomic resolution in HAADF imaging. This microscope also includes a Super-X quad EDX detector for elemental analysis, providing information about atom position and composition simultaneously. Secondary electron (SE) images for the topography study of the TEM specimen were performed using a FEI NOVA NANOSEM 450 microscope at 2 kV.

The Bi-M line at 2.42 keV was used for the quantitative determination of Bi composition through the Bruker Espirit software. The thickness of the specimen was determined from the analysis of the spatially resolved zero-loss EELS signal, by using the Digital Micrograph (GATAN™) software [23]. The column-by-column Bi distribution has been studied using the qHAADF software available to run on the Digital Micrograph. This software allows measuring and mapping the integrated intensity of selected areas around atomic columns by detecting intensity peaks in the HAADF-STEM image [17]. The ω-2θ (0 0 4) XRD spectra were measured with a Bruker D8 Discover X-ray diffractometer using Cu-Kα_1_ radiation. The scans were simulated using Bede Rads Mercury software.

## Results and Discussion

**Fig. 1:**
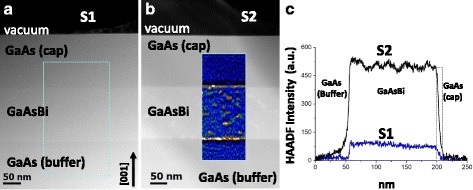
**a** Cross-sectional HAADF-STEM images of sample S1 showing GaAs/GaAsBi/GaAs interfaces. **b** Cross-sectional HAADF-STEM image of sample S2, in the GaAsBi layer bright spots distributed along the GaAsBi layer related to Bi-rich areas are observed. Detail using temperature colour scale of an area after applying a low-pass filter is included as an inset in the same image for a better visualisation. **c** Thickness gradient-corrected intensity profiles taken along [001] direction from the regions marked with green rectangles in the HAADF-STEM images, blue line for sample S1 and black line for sample S2, showing a slightly different behaviour at the interfaces

Figure 1 shows low-magnification HAADF-STEM images taken on [110] zone axis of samples S1 (a) and S2 (b), together with the thickness gradient-corrected intensity profiles taken along the [001] direction from the regions marked in the HAADF-STEM images (green rectangles). No threading dislocations or stacking faults were detected in the studied regions of both samples. In HAADF, the intensity obtained is proportional to the average atomic number. So, for a constant sample thickness, the brighter contrast in the image is related to a higher Bi content (*Z*_Bi_ = 83, *Z*_Ga_ = 31 and *Z*_As_ = 33). This makes possible the study of the Bi distribution in GaAsBi/GaAs heterostructures. As it can be observed in Fig. 1a, no clear contrast variations are detected in the GaAsBi layer in sample S1—with a lower Bi content—not showing clear evidence of clustering, not even in other areas with higher sample thickness. However, Bi and As clusters have been reported in the literature, even for GaAsBi samples with Bi content as low as 1.44%, although grown at lower temperatures [11]. With regard to sample S2, with a higher Bi content, Fig. 1b depicts some areas with brighter contrast in the layer with a relatively homogeneous size and distribution. These regions, regularly distributed along the GaAsBi layer and interfaces, can be directly interpreted as Bi-containing clusters because of their higher HAADF intensity. For a better visualisation, the corresponding low-pass filtered image is shown as an inset in the same image, where the yellow colour corresponds to higher Bi content areas and the black to lower ones (temperature scale). The formation of Bi clusters in GaAsBi without provoking structural defects has been previously reported by other authors [7, 24, 25]. The integration of Bi (1.6 times the atomic volume of As) in the GaAs matrix may cause an increase in the substitutional energy because of the strain, reducing the solubility of the As atoms and allowing the gathering of the Bi ones. A study of the sample using a field emission gun scanning electron microscope (FEG-SEM) was performed to ensure that Bi clusters are embedded within the layer. For that purpose, topography images of secondary electrons acquired at low voltage (not shown here) were compared to STEM ones taken from the same area.

In order to obtain further information on the Bi distribution in the samples, intensity profiles along growth direction with corrected thickness gradient are shown in Fig. 1c. Profiles, taken from the HAADF-STEM images in Fig. 1a, b, point out a similar behaviour in both samples: roughly abrupt interfaces, GaAsBi layers of similar length (~ 140 nm). Regarding the GaAsBi/GaAs interface, the HAADF intensity drops from its maximum value to ~ 0 in about 10 nm (see the grey dashed rectangle in the profile), suggesting some Bi incorporation throughout the GaAs cap layer even without Bi flux. Profiles also depict information about equilibration time. As it can be observed from the profiles, in the lower Bi content sample (S1), the GaAs/GaAsBi interface is more abrupt than that in the higher Bi content sample (S2). This may be explained by the different Bi incorporation coefficients of the two samples. S1, while grown at the same temperature as S2, has a much smaller Bi content. S1 is therefore probably grown under kinetically limited conditions with near-unity Bi incorporation [26], meaning that the Bi surface layer will equilibrate over a time frame less than the surface lifetime of a Bi atom at this temperature. S2, on the other hand, probably has a lower Bi incorporation coefficient [27]. The Bi surface layer in this case would take more than the surface lifetime of a Bi atom to equilibrate, leading to a slower stabilisation of Bi incorporation.

**Fig. 2:**
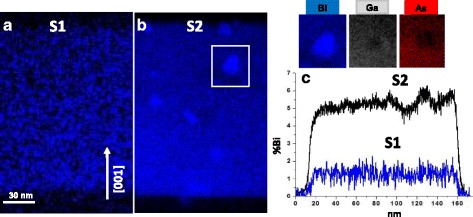
STEM/EDX elemental maps representing Bi distribution in samples S1 (**a**) and S2 (**b**). Details of Bi, Ga and As elemental maps corresponding to the cluster marked with a white rectangle in Fig. 1b reveal a drop in As and Ga signals where there is a high Bi region. **c** Bi content profiles along [001] direction extracted after integration of an area of around 130 nm from the EDX map of samples S1 (blue line) and S2 (black line). Similar features were observed in the intensity profiles at low magnification shown in Fig. 1c

To confirm the correlation made between the HAADF-STEM intensity profiles and the Bi distribution in the heterostructure, STEM-EDX Bi compositional maps of the samples were simultaneously taken. They are shown in Fig. 2 for sample at low (a) and high (b) Bi flux. The corresponding Bi compositional profiles along the growth direction, determined by integrating the point EDX spectra of an area of around 130 nm, are shown in Fig. 2c as blue and black lines respectively. These compositional profiles display the same tendency detected in the GaAs/GaAsBi/GaAs interfaces by low-magnification HAADF analysis. The average Bi atomic fraction in the GaAsBi layers was quantified from the corresponding EDX spectra being1.2 ± 0.4% and 5.3 ± 0.4% in samples S1 and S2, respectively, showing a non-homogeneous distribution of Bi in the GaAsBi layer in both samples.

The presence of Bi clusters would be due to a steric hindrance effect. In this case, the surface tension may increase because of the larger atomic size of the Bi atoms, so to reduce the stress in the structure Bi atoms could be blocking Ga incorporation and consequently causing Ga vacancies in the net. Ga and As compositional maps of the cluster surrounded by a white square in Fig. 2b are included to show how both signals fall where there is a high Bi signal. This suggests that, in this particular cluster, Bi could be occupying both group III and group V sub-lattices. This also evidences that the clusters are not superficial ones formed during TEM sample preparation.

To carry out a deep study of the Bi distribution at the atomic level, high-magnification aberration-corrected HAADF-STEM images were taken in the [110] projection. In this III–V semiconductor alloy, the two maximum intensity peaks of a dumbbell correspond to group III and group V atomic columns. To properly correlate the intensity in the atomic columns with their composition, the background level has been removed from the experimental HAADF-STEM images. Then, an automatic location of the intensity peaks has been carried out, and integration areas around the group V atomic columns are carefully selected. Integrated intensities are measured and mapped for every dumbbell using the qHAADF approach. The procedure to quantify Bi content was similar to the one published in Ref. [28]. In this work, the integrated intensity quotients of every dumbbell (***R***) was calculated as a ratio between the integrated intensity in the group V columns (*I*_As − Bi_) in the whole image and the mean integrated intensity in the group V columns in the GaAs layer (*I*_As_), as *R* = (*I*_(As − Bi)_)/*I*_As_.

**Fig. 3:**
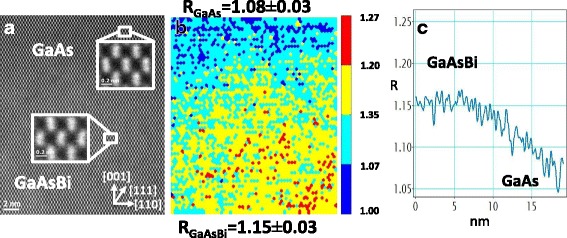
**a** Cross-sectional [110] HAADF-STEM image of the interface GaAsBi/GaAs of sample S1. The detail of an undistorted region in the GaAs layer and distorted anion-cation dumbbells in a Bi-rich area at higher magnification are included as an inset in the same image. **b** Coloured map representing *R* values around group V columns of the HAADF image in Fig. 3a. The green colour corresponds to mean Bi composition in GaAsBi layer measured by EDX. Despite the homogeneous distribution of Bi in the HAADF-STEM image, the intensity map shows areas with high probability of nanoclustering (red dots) and columns with relative poor Bi content (blue dots) in the GaAsBi layer. **c** Profile along the growth direction of the whole *R* intensity map, showing a blurred GaAsBi/GaAs interface in about 10 nm

Figure 3a shows a high-magnification HAADF-STEM image of the GaAs/GaAsBi interface taken from the sample with poor Bi content (S1). The coloured normalised integrated intensity map of the HAADF image is shown in Fig. 3b. Coloured dots ranging from 1 (deep blue) to 1.27 (red) depict the Bi content in the group V columns. As it can be observed, small fluctuations in *R* values from the mean value are found in both layers. In order to compare the dispersion of the obtained results, we have calculated the correlation coefficient ***Cv*** (defined as the ratio between the standard deviation and the mean *R* value) in the GaAs (substrate), the GaAsBi layer and the GaAs cap layer. The ***Cv*** values were 1.3, 2.6 and 2.6%, respectively, as fluctuations observed in GaAsBi/GaAs interface are higher than those found in the GaAs substrate taken as a reference; we can consider that these variations in *R* values are related to changes in Bi column composition. The cause of the non-zero ***Cv*** factor in the substrate may be due to local thickness fluctuations, detector noise (measured in a region without material as 0.6%) or hydrocarbons being deposited on the surface sample during microscopy characterisation.

This qHAADF analysis confirms a non-abrupt upper GaAsBi/GaAs interface over about 10 nm perceived at low magnification, mainly due to Bi surface segregation during growth, as it can be observed from the profile taken along the growth direction in the whole intensity *R* map, shown in Fig. 3c. Furthermore, dispersed Bi-rich columns within the GaAsBi layer (red dots), together with areas with a poor Bi content (blue dots) in the GaAsBi layer, are detected as well with this software, confirming the Bi content fluctuations in the GaAsBi layer. The presence of Bi-rich columns seems to be producing distinct distortion in the dumbbell shape as it can be appreciated in the inset at higher magnification in Fig. 3a. The substitution of As atoms for bigger Bi ones during the epitaxial growth would have locally broadened the lattice of the matrix, causing a distortion in the dumbbell shape while the structure is maintained.

HAADF-STEM techniques also enable structural and compositional analysis of the clusters detected at low magnification in sample S2. These clusters, almost homogeneously distributed, occupy around 20–30% of the GaAsBi layer surface. To know Bi composition, shape and size of the clusters found, aberration-corrected HAADF-STEM images were taken in the [110] projection and EDX elemental maps and intensity map ratios around group V columns were performed. To identify different crystalline phases, a fast Fourier transform (FFT) study was carried out in the high-resolution images in different areas inside and nearby the clusters.

**Fig. 4:**
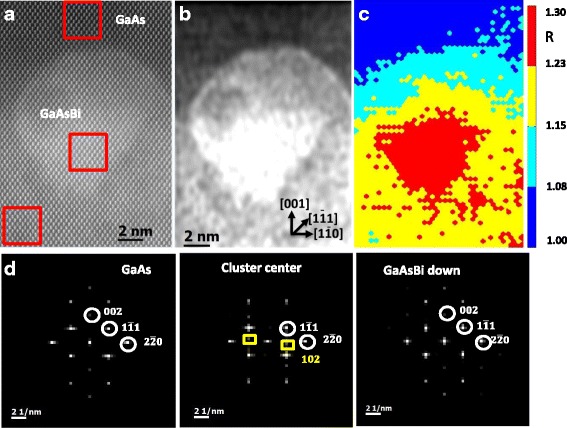
**a** Cross-sectional [110] HAADF-STEM image of the interface GaAsBi/GaAs of sample S2, capturing a Bi cluster of about 12 nm in size, next to the GaAsBi/GaAs interface. **b** Low-filtered image of the HAADF-STEM image shows two areas with different contrast in the GaAsBi layer, a Bi-rich zone is faceted along {111} and (001) planes, surrounded by a lower Bi-rich one. **c** Coloured map representing the *R* values around group V columns depicting a graded Bi distribution around the cluster. **d** The corresponding Fourier transformation from the selected areas marked with red rectangles in Fig. 1a. Additional spots matching {102} planes related to rh-Bi phase are detected in the highest contrast cluster region

Figure 4a shows a high-resolution [110] HAADF-STEM image of the GaAsBi/GaAs interface with a clear Bi cluster of about 12 nm diameter. Red rectangles in the image represent the areas where the FFT study was performed. It is well known that low-pass filtering not only reduces the amount of noise in the data, but it also removes the periodic features observed in the raw image, emphasising the edges of the Bi cluster. The filtered HAADF-STEM image, following the procedure described by Werner et al. [29], is shown in Fig. 4b. As it can be appreciated, {111} and (001) facets in the brightest area are clearly observable. However, the intensity distribution suggests the presence of two different compositional volumes in the particle: a Bi-rich one with trapezoidal shape surrounded by a less Bi-rich area. A similar cluster shape was observed for Wood et al. in a five-period GaAs_1 − *x*_Bi_*x*_/GaAs_1 − *y*_Bi_*y*_ structure [10].

The qHAADF analysis through the intensity ***R*** map shown in Fig. 4c depicts a roughly equiaxial particle shape and a Bi concentration gradient peaked at the centre of the cluster.

Wu et al. [4] reported the presence of different crystallographic structures in GaAsBi layers studying HRTEM micrographs and modelling the formation and the phase transformation from zinc blende Bi-rich to rhombohedral Bi (rh-Bi) nucleated in zinc blende {111} planes. In this sense, it is worth mentioning that bright spots in the FFT from high-resolution HAADF-STEM images can be interpreted as diffraction spots from crystallographic planes. Then, extra spots in the conventional pattern for the zinc blende structure of GaAs should be interpreted as additional phases. To study the presence of different crystal structures in the cluster, Fig. 4d shows the FFTs corresponding to three different areas marked as red squares in Fig. 1a. From right to left shows a homogeneous region in GaAs cap layer, the brightest zone in the GaAsBi layer and another region with lower contrast in the same GaAsBi layer. White circles denote the position of diffraction peak with Miller indices 002, $1\overline{1}1$ and$2\overline{2}0$. As it can be observed, only in the centre of the cluster appears clear spots, marked with a yellow square, related to {102} planes roughly parallel to zinc blende {220} planes, suggesting that a new rh-Bi phase has nucleated in the GaAsBi zinc blende layer. FFTs also depict a broadening of the diffraction peaks related to {111} planes. This observed asymmetric peak may be due to the micro-strain causes by shearing {111} planes between rh-Bi and zinc blende phases in the cluster.

For a deep study of cluster composition, it is worth mentioning that when electrons go through an electron transparent specimen with embedded clusters, they carry information not only about the cluster, but also from the matrix. So, to estimate real cluster composition, we follow the same procedure described in Ref. [25]. Results also pointed out that clusters close to the GaAsBi/GaAs interface tend to be smaller in size (12 nm) with a higher Bi content (≈ 30%), probably due to the presence of Bi on the surface when the cap layer begins to be grown. However, most of the clusters located inside the GaAsBi layers have a bigger size (16 nm), but their Bi content is inferior (≈ 22%). Additionally, in the GaAsBi layer, clusters with superior Bi content (35%) and higher size (23 nm) were detected. Additionally, as it was mentioned before, the core of some clusters consist of pure rh-Bi.

**Fig. 5:**
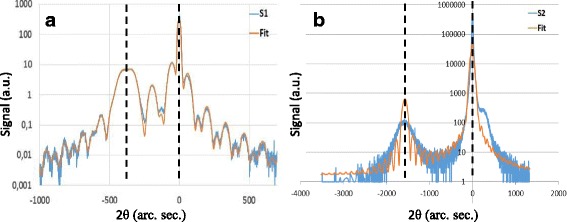
X-ray diffraction data (blue lines) and simulated fits (orange lines) of sample S1 (**a**) and sample S2 (**b**). Vertical dashed lines highlighting the GaAs peak at 0° arc seconds and the GaAsBi film peak located at negative arc seconds. The shoulder on the right GaAs peak in Fig. 5b is indicative of a GaAs cap layer under tensile strain

In order to investigate the strain state of the epitaxial film, high-resolution X-ray ω-2θ curves were recorded. Figure 5 shows the (004) XRD scans of the GaAsBi/GaAs layers in blue and simulated fits in orange for samples S1 (a) and S2 (b). In both samples, the sharpest and highest intensity peak located at 0° arc seconds corresponds to the GaAs substrate, while the broad lower intensity peak located at negative arc seconds corresponds to the strained GaAsBi layer. The angle between the peaks relates to the amount of lattice mismatch between both layers. The shoulder to the right of the GaAs peak in the spectrum of S2 indicates a GaAs layer under tensile strain; this implies strain relaxation in the S2 GaAsBi layer. The XRD spectrum of sample S1 was well fitted using the Bi fraction and thicknesses given by the EDX and HAADF measurements. There is no indication of any strain relaxation in the XRD spectrum of sample S1. The XRD spectrum of sample S2 was more problematic to fit. Figure 5b shows the data modelled by a uniform GaAsBi layer of 5.8%, as determined by the HAADF-STEM analysis, ignoring the Bi rich clusters, and a relaxation of the GaAsBi layer of 6%, as determined by fitting the XRD curve; this would be reasonable if the clusters were not coherent with the rest of the GaAsBi matrix. This model does not accurately represent the layer; while the substrate-GaAsBi splitting is sufficient to account for the data, no tensile GaAs peak at ~ 250° arc seconds is visible. The tensile GaAs peak suggests that relaxation has occurred within the layer. Relaxation of a compressive layer will reduce the out of plane lattice constant, which implies that the modelled average Bi content of 5.8% is an underestimate of the real average; this is consistent with the observation of Bi-rich clusters in the layer. No peak(s) corresponding to ~ 22–35% GaAsBi was observed by reciprocal space mapping (not shown), which suggests that the clusters may not be coherent with the GaAsBi matrix. No further modelling of the S2 XRD spectrum was attempted.

## Conclusions

The ac-HAADF-STEM analyses offer useful information about structure and composition of GaAs/GaAsBi/GaAs heterostructures, the results being in good agreement with EDX, FFT and XRD investigations. The analysis of low-magnification HAADF-STEM images allowed detecting inhomogeneous Bi distribution and non-abrupt GaAsBi/GaAs interfaces. At high magnification, the qHAADF analysis revealed clear Bi segregation at the GaAsBi/GaAs interface at low Bi flux (S1), and distorted dumbbell shape in areas with higher Bi fraction, due to As-substitutional positions of Bi atoms in the group V subnet. At higher Bi flux (S2), the size of the Bi gathering raises leading to roughly equiaxial clusters uniformly dispersed around the whole matrix and interfaces. The study revealed the coexistence of two different crystalline phases in the studied clusters rh-Bi and zinc blende shearing {111} planes, affecting to the local lattice relaxation and leading to a partially relaxed GaAsBi/GaAs system, in good agreement with XRD analysis.

## Abbreviations

Ac-HAADF-STEM: Aberration-corrected high-angle annular dark-field scanning transmission electron microscopy
EDX: Energy-dispersive X-ray
EELS: Electron energy loss spectrum
ESR: Conventional electron spin resonance
FEG: Cold field emission gun
FFT: Fourier fast transform
HRTEM: High-resolution transmission electron microscopy
IMEYMAT: Instituto Universitario de Investigación en Microscopía Electrónica y Materiales
MBE: Molecular beam epitaxy
qHAADF: Quantitative HAADF image analysis algorithm
SEM: Scanning electron microscope
XAS: X-ray absorption spectroscopy
XRD: X-ray diffraction
